# Aeroallergen sensitization rate and its multifactorial influences in continental Croatia: a cross-sectional study

**DOI:** 10.3325/cmj.2025.82

**Published:** 2025-04

**Authors:** Vesna Vukičević Lazarević, Ivan Marković

**Affiliations:** Special Hospital for Pulmonary Diseases, Zagreb, Croatia

## Abstract

**Aims:**

To assess the rate of sensitization to airborne allergens in continental Croatia and the related variables. A secondary aim was to assess the frequency of pollen-food syndrome.

**Methods:**

This cross-sectional study enrolled 2133 participants referred to Special Hospital for Pulmonary Diseases, Zagreb, from January 2 to December 31, 2022. Patients underwent skin prick test, and data on age, area of residence, smoking status, referring physician, and the presence of respiratory disease were gathered from electronic health records.

**Results:**

The rate of sensitization in our sample was 48.86%, and that of polysensitization was 75.91%, with grass pollen being the predominant allergen. Sensitization rates to all airborne allergens significantly increased compared with a 2003-2006 cohort. Men had higher rates of sensitization, and sensitivity to certain allergens varied based on age. Patients diagnosed with rhinitis exhibited the highest rates of sensitization and mostly sought medical attention from allergists. Conversely, asthma patients who did not have rhinitis exhibited reduced rates of sensitization and were mostly treated by pulmonologists. Birch tree pollen was the primary aeroallergen responsible for pollen-food syndrome, regardless of the age group.

**Conclusions:**

Our findings contribute to the existing body of research on sensitization to aeroallergens in Europe and stress the importance of multidisciplinary management of allergic respiratory disorders.

Around 10%-30% of the global population has allergic diseases, with the rates varying across countries (1-3). Two decades ago, a Croatian study found high sensitivity to grass pollen (46.91%), ragweed (42.07%), birch pollen (25.56%), house dust mites (HDM) (36.45%), and animal dander (5.1%) (4,5). However, in recent years, global allergy expansion has been a major concern (6), especially that of airway allergies, whose prevalence in Europe increased from 23 to 31% (7). Germany has seen a surge in practically all aeroallergen sensitization rates (8). Sensitization to grass and rye pollen was highest, with an increased sensitization to HDM in symptomatic individuals (8). Like Germany, northern continental Croatia is geographically and climatologically positioned in central Europe; therefore, similar results are anticipated.

In 2022, Topalušić et al (9) found a considerable rise in allergic rhinitis and atopic dermatitis prevalence among Croatian children, whereas asthma prevalence remained stable. Child allergen sensitivity has remained the same, but grass pollen has become the dominating allergen.

Croatian adults have not been examined for pollen food allergy syndrome (PFS). However, its prevalence in the world varies widely by area, from 2% to 70% (10).

The recent ARIA-MeDALL theory (11) challenged the “one-airway-one-disease” concept (12) and introduced the hypothesis of multimorbid allergic disease. According to this theory, rhinitis alone is a local disease, but rhinitis with asthma is a systemic disease, with the microbiome being the modulating factor (11). Based on this concept, we aimed to investigate whether patients with rhinitis alone, asthma alone, or both rhinitis and asthma exhibited different patterns of allergen sensitization.

Most recent studies on allergic sensitization patterns, changes in prevalence and their association with respiratory diseases in Europe have not included patients from Croatia. As a result, trends and changes occurring in our population remain unclear. The aim of the study was to assess the rate of inhaled allergen sensitivity in Croatian adults and its related variables (age, place of residence, smoking habits, and respiratory diseases). Additionally, we included the variable “referring physician” to explore potential differences in patient profiles between referrals from pulmonologists, allergists, and general practitioners, and to assess the need for improved collaboration among these specialties. The study also examined patient characteristics based on their referring physicians, and PFS rate and sensitization profiles in patients with PFS.

## Patients and methods

### Patients

This cross-sectional study enrolled adult patients referred to the Special Hospital for Pulmonary Diseases in Zagreb, Croatia. The patients were referred to our center for skin prick test (SPT) by general practitioners (GPs) or other physicians working in our hospital: pulmonologists, allergists, and immunologists. The study was approved by the Ethics Committee of the Special Hospital for Pulmonary Diseases.

### Methods

This study enrolled patients who underwent SPT at our clinic from January 2022 to January 2023. Demographic data, smoking status, and medical history if available were obtained retrospectively from hospital electronic health records (EHRs).

SPT was conducted using 16 aeroallergen solutions (Diater, Barcelona, Spain), with positive control (histamine hydrochloride 10 mg/mL) and negative control (diluent) solutions applied on the volar side of the forearm. A positive result was defined as exceeding a threshold of 3 mm. The aeroallergen spectrum comprises various substances, including cat dander (e1), dog dander (e5), *Dermatophagoides pteronyssinus* (d1), *Dermatophagoides farinae* (d2), alder (*Alnus glutinosa)* (t2), birch (*Betula verrucosa*), hazel (*Corylus avelana*) (t3), plane tree (*Platanus acerifolia*) (t11), poplar (*Populus deltoides*) (t14), ash (*Fraxinus excelsior*) (t25), Bermuda grass (*Cynodon dactylon*) (g2), orchard grass (*Dactylis glomerata*) (g3), Timothy grass (*Phleum pratense*) (g6), common meadow grass (*Poa pratensis*) (g8), maize (*Zea mays*) (g202), ragweed (*Ambrosia arte*) (w1), and mugwort (*Artemisia vulgaris*) (w6).

The results of d1 and d2 were merged into an HDM-positive group, g2, g3, g6, g8, and g202 into a grass-positive group, and t2, t3, and t4 into a birch tree family-positive group. Other allergens were analyzed as separate allergens. Patients sensitized to only one allergen or a group of allergens were considered monosensitized, while patients sensitized to two or more individual or group allergens were considered polysensitized.

For patients referred by GPs, we collected data on age, sex, and SPT results. For patients referred by specialists working at our institution, we also obtained the data on the concurrent medical conditions, smoking habits, and the presence of respiratory diseases (RD).

Specialists working at our institution established the diagnoses of RD as a part of routine clinical practice according to the Global Initiative for Asthma (13) and the Allergic Rhinitis and Its Impact on Asthma initiative (14) guidelines. RD were categorized into three groups: rhinitis without asthma, rhinitis with asthma, and asthma without rhinitis. The aim was to identify differences in allergen sensitization patterns among the three clinical conditions, in line with the ARIA-MeDALL hypothesis (11). Patients diagnosed with chronic obstructive pulmonary disease according to the GOLD guidelines (15) but without any overlap with asthma were classified as having other conditions.

**Figure 1:**
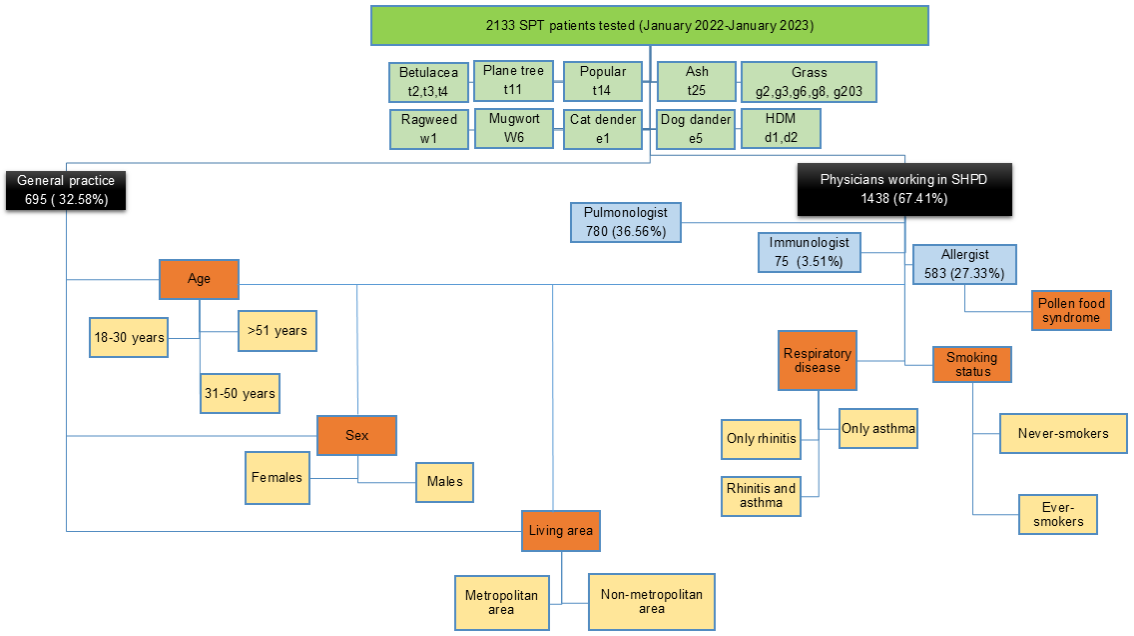
The process of data collection. SPT – skin prick test; SHPD – Special Hospital for Pulmonary Diseases.

The PFS data were acquired solely from patients referred by allergists, as this type of data is rarely included in other physicians' reports. The PFS diagnosis was based on the current BSACI guidelines (16). The data collection procedure is illustrated in Figure 1.

The patients were stratified into three age groups: 18-30 years, 31-50 years, and 51 years and above. They were also categorized into two groups according to the area of residence: the Zagreb metropolitan area and non-metropolitan area. The smoking status was categorized into two groups: 1) ever-smokers, individuals who were actively smoking at the time of the study and individuals who had previously smoked but had since quit; and 2) never-smokers, individuals who had never smoked. Smoking is usually meticulously recorded in reports by physicians working in our hospital; if the smoking status was not evident from EHR, we assumed the patient was a never-smoker.

### Statistical analysis

The Shapiro-Wilk test was used to determine the normality of distribution of continuous variables. The significance of differences in categorical variables was tested with a χ^2^ test of independence. The significance of differences in continuous variables was tested with a student *t* test or analysis of variance (ANOVA), with a *post-hoc* Tukey's HSD test. Patients with missing or incomplete data were not included in the study. A *p*-value lower than 0.05 was considered statistically significant. The Bonferroni correction was applied to correct for multiple comparisons, with significance set at *P* < 0.0125. The statistical analysis was performed with JMP® Pro 16 (SAS Institute Inc., Cary, NC, USA).

## Results

The study enrolled 2133 patients, mostly women (67.27%) with a mean age of 46.13 years. The majority (67.08%) of the participants lived in the Zagreb metropolitan area. Almost half (48.86%) tested positive for inhaled allergens, with the majority being polysensitized (Table 1). Most of the patients were sensitized to grass, followed by ragweed, birch tree family, HDM, ash, cat and dog dander, mugwort, plane tree, and poplar (Table 2).

**Table 1:** Demographic characteristics of adults included in the study

	No. (%) of participants			
Variable	whole cohort	female	male	*P* value*
**Number of participants** (%)	2133	1435 (67.27)	698 (32.72)	
**Age** mean (median, range)	46.13 (45,18-89)	47.10 (47, 18-89)	44,13 (42, 18-85)	<0.001
**Area of residence**				
Metropolitan area	1431 (67.08)	991 (69.05)	440 (63.03)	
Non- metropolitan area	702 (32.91)	444 (30.94)	258 (36.96)	0.005
**Any sensitization**	1038 (48.86)	621 (43.27)	417 (59.74)	<0.001
**Polysensitization** **N(%)**	788 (75.91)	453 (72.94)	335 (80.33)	0.006

*χ^2^ test for categorical variables or *t* test for the continuous variables.

**Table 2:** The rates of sensitization to different inhaled allergens

Sensitizing allergens	No. (%)
**Grasses**	613 (59.05)
Meadow grass (*Poa pratensis*)	524 (85.48)
Timothy grass (*Phleum pratense*)	498 (81.23)
Orchard grass (*Dactylis glomerata*)	457 (74.51)
Bermuda grass (*Cynodon dactylon*)	390 (63.62)
Maise (*Zea mays*)	356 (58.07)
**Birch tree family**	564 (54.35)
Birch (*Betula verrucosa*)	510 (90.42)
Alder (*Alnus glutinosa*)	489 (86.70)
Hazel (*Corylus avelana*)	476 (84.39)
**Ash**	314 (30.25)
**Poplar**	173 (16.60)
**Plane tree**	212 (20.42)
**House dust mite**	485 (46.72)
*Dermatophagoides pteronyssinus*	463 (95.46)
*Dermatophagoides farinae*	431 (88.86)
**Cat dander**	291 (28.03)
**Dog dander**	259 (24.95)
**Ragweed**	586 (56.45)

The majority of monosensitized patients were sensitized to HDM (84, 33.6%), followed by ragweed (54, 21.6%), the birch tree family (39, 15.6%), grasses (37, 14.8%), cat dander (11, 4.4%), dog dander (10, 4%), ash (8, 3.2%), poplar (3, 1.2%), plane tree (2, 0.8%), and mugwort (2, 0.8%).

**Figure 2:**
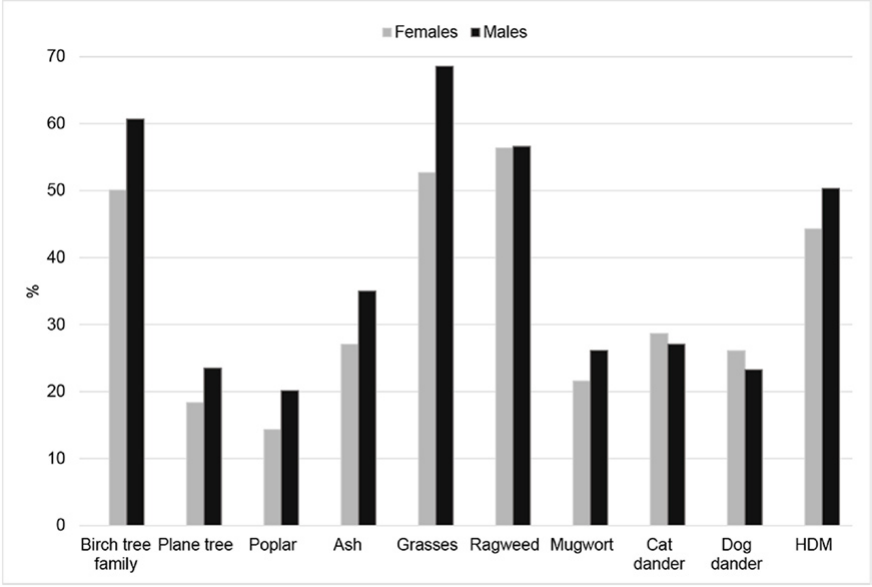
Differences in the sensitization to studied allergens according to sex. HDM – house dust mites.

Significantly more men than women were sensitized (59.74% vs 43.27%, *P* < 0.001), but fewer men than women were monosensitized (19.66% vs 27%; *P* = 0.006). Specifically, men were significantly more sensitized to the birch tree family (*P* < 0.001), plane tree (*P* = 0.043), poplar (*P* = 0.013), ash (*P* = 0.006), and grasses (*P* < 0.001) (Figure 2).

**Figure 3:**
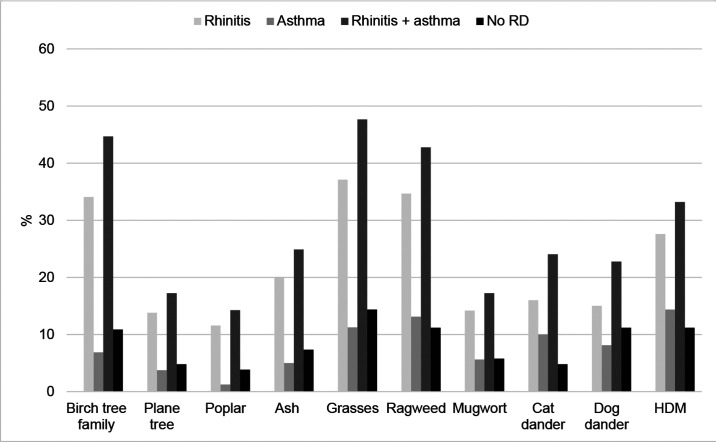
Differences in the sensitization to studied allergens according to the presence of respiratory conditions (rhinitis vs asthma vs rhinitis plus asthma). HDM – house dust mites.

Our hospital doctors referred 1438 patients for SPT, 1123 of whom were diagnosed with RD. The majority of them had rhinitis alone, followed by those with both rhinitis and asthma, while the smallest proportion had asthma alone (Table 3). Patients who had asthma without rhinitis were significantly older than patients with rhinitis with and without asthma. A significantly higher rate of sensitization to inhaled allergens was found in patients with rhinitis with and without asthma compared with patients with only asthma and without RD. Moreover, the highest polysensitization rate was observed in patients with both rhinitis and asthma (Figure 3). Sex did not affect the rate of RD, while patients who smoked had the highest rate of only asthma.

**Table 3:** Sociodemographic characteristics and sensitization rates in patients with and without respiratory disease

	Whole cohort	No. (%) of patients				*P* value*
Variable		with respiratory disease (RD) N = 1123			without RD	
		rhinitis without asthma	asthma without rhinitis	rhinitis and asthma		
**No.**	1438	493	160	470	315	
**Age** mean (median, range)	44.84 (48, 18-87)	44.07 (43, 18-81)	56.18 (60, 19-87)	46.04 (46, 18-85)	52.22 (53, 19-85)	<0.001
**Sex**						
women	940 (65.36)	318 (64.15)	111 (69.37)	293 (62.34)	218 (69.20)	0.120
men	498 (34.63)	177 (35.90)	49 (30.62)	177 (37.66)	95 (30.15)	
**Ever –smokers**	490 (34.07)	137 (27.79)	89 (55.62)	146 (31.06)	118 (37.65)	<0.001
**Metropolitan** **residence**	968 (67.31)	339 (68.76)	102(63.75)	317(67.44)	210(66.66)	0.733
**Any sensitization**	736 (51.11)	284 (57.60)	53 (33.12)	323 (68.72)	76 (24.12)	<0.001
**Polysensitization**	575 (78.12)	221 (77.81)	32 (60.37)	276 (85.44)	46 (58.97)	<0.001

*χ^2^ test for categorical variables and ANOVA-t for the continuous variables. *P* < 0.0125 is considered significant when Bonferroni-corrected.

GPs referred 695 (32.58%), allergists 583 (27.28%), immunologists 75 (3.51%), and pulmonologists 780 (36.56%) patients. Rhinitis was considerably more common in patients referred by allergists and immunologists, whereas asthma with or without rhinitis was more common in patients referred by pulmonologists (Table 4). Patients treated by allergists and immunologists were approximately ten years younger than those treated by pulmonologists. Patients referred by allergists and immunologists had higher rates of positive inhaled allergen testing than those referred by pulmonologists or GPs (59.69% vs 58.67% vs 44.10% vs 43.45%, respectively, *P* < 0.001). They also had significantly higher polysensitization rates: 81.90% vs 81.82% vs 74.42% vs 69.87%, respectively (*P* = 0.002).

**Table 4:** The type of referring physician in patients with different respiratory diagnoses (N = 1438)

Variable	No. (%) of patients treated by			
	allergist N = 583 (27.28)	immunologist N = 75 (3.51)	pulmonologist N = 780 (36.56)	P -value
**Allergic rhinoconjuctivitis**	278 (47.85)	30 (40)	185 (23.72)	<0.001
**Asthma**	20 (3.44)	1 (1.33)	139 (17.82)	<0.001
**Allergic rhinoconjuctivitis + asthma**	149 (26.65)	15 (20)	306 (39.73)	<0.001
**Other**	134 (23.06)	29 (38.67)	150 (19.3)	<0.001
**Age, mean** **(range)**	41.8 (18-82)	42.1 (19-79)	52.9 (19-87)	<0.001

*χ^2^ test for categorical variables and ANOVA for the quantitative continuous variables.

Our study compared sensitization rates and overall sensitivity among three age groups. These groups significantly differed (*P* < 0.001) in both overall sensitization and monosensitization. The highest percentage of sensitized patients was found in the age group 18-30, with 66.93% (328/490) of patients, followed by the group aged 31-50, with 53.48% (414/774) of patients, and the group aged 51+, with 34.06% (296/869) of patients.

**Figure 4:**
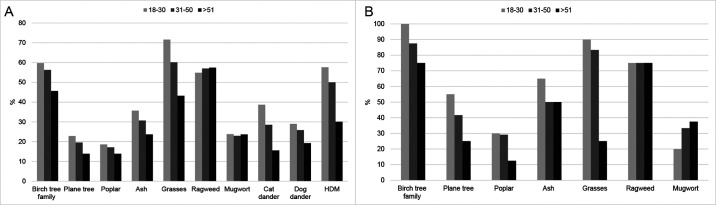
Differences in the sensitization to studied allergens according to age group (**A**) in the entire sample; (**B**) among patients with pollen food syndrome. HDM – house dust mites.

The lowest percentage of monosensitized patients was in the 18-30 age group (16.15% or 53/328), followed by the 31-50 age group (22.22% or 92/414) and the 51+ age group (35.47% or 105/296). Patients aged 18-30 and 31-50 had significantly higher (*P* < 0.001) rates of HDM monosensitization (47.16% and 44.56%, respectively) than those aged 51+ (17.14%). In contrast, the 51+ age group had a significantly higher rate of ragweed monosensitization (39.09%) than the 18-30 age group (3.77%) and the 31-50 age group (11.95%) (*P* < 0.001). Monosensitization rates for other allergens were similar across all age groups. Furthermore, the three age groups also significantly differed in the overall sensitization to grasses (*P* < 0.001), the birch tree family (*P* = 0.001), ash (*P* = 0.004), cat dander (*P* < 0.001), dog dander (*P* = 0.017), and HDM (*P* < 0.001) (Figure 4A).

PFS rates were only available from allergists’ reports. Rhinitis patients with or without asthma had a 12.64% rate of PFS, with 96.15% being polysensitized. The two monosensitized individuals were 51 or older and positive for the birch tree family (*P* = 0.003). Most PFS patients (n = 47) tested positive for Betulaceae (90.30%), 40 for grass (76.26%), 39 for ragweed (75%), 29 for ash (55.76%), 23 for plane tree (44.23%), 52 for mugwort (28.84%), and 14 for poplar (26.92%). The three age groups did not significantly differ in PFS rate (*P* = 0.8271) and sensitization to particular allergens, except for grasses (*P* = 0.002), which was lower in the 51+ age group (Figure 4B).

**Figure 5:**
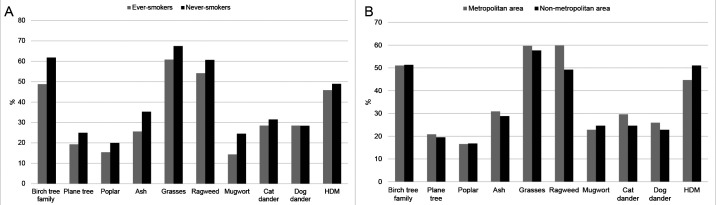
Differences in the sensitization to studied allergens according to (**A**) smoking history (ever-smokers vs never-smokers) and (**B**) the area of residence (metropolitan vs nonmetropolitan). HDM – house dust mites.

Of 1438 patients having smoking status data, 490 (34.07%) were ever-smokers and 948 (65.92%) were never-smokers. There were significantly more male smokers (38.35% to 31.80%; *P* = 0.018). Never-smokers were more sensitive to inhaled allergens (54.95% vs 28.43%) than ever-smokers (*P* < 0.001). The results were consistent for all allergens except dog dander (Figure 5A).

The majority of patients (67.13%) lived in the metropolitan area. The overall sensitization to the tested allergens was similar between metropolitan and non-metropolitan patients (49.26% and 47.43%, respectively; *P* = 0.426), but ragweed sensitization was significantly higher in metropolitan residents (59.85% vs 49.23%; *P* = 0.002) (Figure 5B). Monosensitization and polysensitization rates did not vary based on the residence area (*P* = 0.974).

## Discussion

This study showed high sensitization and polysensitization rates, with pollen (grasses) being the most common allergen. Men had a higher incidence of sensitization and polysensitization, with no discernible influence of sex on the prevalence of RD, as seen in previous studies (17-20). Furthermore, similarly to previous studies (21,22), the age group 18-30 had the highest overall sensitization and polysensitization rates. Most monosensitized patients were sensitized to HDM, especially those younger than 51 years old, while patients 51 and older were mainly monosensitized to ragweed.

Our research cohort had a high rate of RD since most patients were referred to SPT for RD symptoms. However, we found no significant difference in the measured variables between individuals with rhinitis alone and those with rhinitis and asthma, which supports the “one-airway-one-disease” concept (12).

Our patients with rhinitis, the most common respiratory condition, had the greatest sensitization and polysensitization rates. The youngest, most sensitized individuals were referred to SPT by allergists. Pulmonologists mostly treated patients with asthma without rhinitis, the least frequent respiratory condition in our sample, who had the lowest sensitization and polysensitization rates. These findings match earlier research (23). Non-allergic asthma is a more severe (24,25) form of asthma and is usually treated by pulmonologists (23).

The high percentage of patients with rhinitis and asthma who were not sensitized to any of the tested allergens may be explained by the sensitization to allergens not in our panel (eg, molds, other pollens) or other causes of RD (eg, vasomotor rhinitis, exercise-induced asthma, non-allergic asthma) (13,14) or smoking (26).

The PFS rate was similar to the most recent data from Turkey (27), and was the same across all age groups, with the majority of patients being sensitive to the birch tree family, even though grass was the most common sensitizer in the entire studied group. This makes birch tree pollen a clear primary sensitizing aeroallergen, as in other central European countries (28).

Most of our patients were never-smokers and had much higher sensitivity to all evaluated allergens, except for dog dander, than ever-smokers. Finally, most of our patients lived in a metropolitan region. However, the area of residence did not affect sensitization and polysensitization rates except for ragweed, which was higher in the metropolitan group. Previous studies linked urban living to increased sensitivity (29,30). We believe this was due to a positive net population migration balance in Zagreb from non-metropolitan regions (31), which confounds our adult population findings. The Zagreb metropolitan region has plenty of ragweed pollen (32). However, a decreasing east-to-west gradient of ragweed pollen in continental Croatia around Zagreb (33) could explain why Zagreb residents are more ragweed sensitive.

Comparable research applied similar methods (4,5,21,34,35), which makes it easier to compare the results. The Galen studies (35,36) explored sensitization tendencies in various European nations excluding Croatia. The most prevalent sensitizers in Europe were grasses, followed by HDM, the birch tree family, cat and dog dander, ragweed, and mugwort, with rates varying across the studied regions (36). This study represents the second investigation into sensitization patterns in the adult population of continental Croatia. A prior study involving the Croatian adult population conducted from 2003-2006 (4) reported notable rates of sensitization to pollen, mites, and spores. This previous study enrolled fewer smokers (20.39% vs 34.07%), but more men (44.34% vs 32.33%) and metropolitan area residents (77.73% vs 67.08%) compared with our study. A comparison of our 2022 cohort to theirs (4) revealed a significant increase in sensitization rates to all aeroallergens. Although the rates of monosensitization and the two most prevalent allergens (HDM and ragweed) responsible for it have remained stable over the past twenty years, our study group, consisting of younger individuals, exhibited increased sensitizations among patients with multiple allergies. Similar results were found in another study that evaluated changes in sensitization to common aeroallergens over the same period (21).

In comparison with a study in Croatian children (9), our adult cohort exhibited similar sensitization rates to grasses, ragweed, and HDM but higher sensitization rates to the birch tree family and cat dander. Our data support the findings of previous research that found a higher burden of birch allergy and PFS in adults than in children in areas with many birch trees (37), with different molecular patterns of sensitization to birch (38). Moreover, in recent decades hypersensitivity to tree pollen, especially birch pollen, has increased among children with asthma (39).

Our research has several limitations. It was conducted at a single institution, which makes our findings potentially not applicable to a more diverse population. The reliance on EHR introduces possible biases due to gaps and inaccuracies in documentation. The lack of precise information, including the reason for referral and other clinical data, may limit the accuracy of information for GP-referred patients. However, this restriction applies only to extra clinical background and does not affect our primary aim of assessing sensitization rates to various inhaled allergens in SPT-positive patients.

Further multicentric prospective longitudinal studies should be performed to increase the generalizability and representativeness, as well as to track sensitization trends and respiratory illnesses over time. Additionally, participants should be tested for other pollens, animal dander, and mold allergies. More detailed sensitization patterns may reveal further respiratory issues. Sensitization trends in different parts of Croatia should also be examined to uncover differences and understand the effects of the continental and Mediterranean climates. Research should evaluate the influence of allergen avoidance and allergy immunotherapy on sensitization and respiratory disorders. Finally, further research is needed on the underlying mechanisms of sensitization and to evaluate the effectiveness of different treatment approaches in managing allergic respiratory diseases.

In conclusion, the findings of this study contribute to the existing body of research on sensitization to aeroallergens in Europe. The high rates of sensitization and polysensitization observed in the study population highlight the importance of addressing allergic respiratory diseases in clinical practice. The predominance of grass pollen as the most common allergen aligns with previous studies conducted in Croatia and Europe. The age-related differences in sensitization patterns suggest that age should be considered when assessing and managing sensitization in patients. The health care utilization patterns of patients with rhinitis and asthma without rhinitis indicate the need for multidisciplinary care involving both allergists and pulmonologists.

## References

[1] Ring J Akdis C Behrendt H Lauener RP Schäppi G Akdis M Davos Declaration: Allergy as a Global Problem. Allergy 2012 67 141 3 10.1111/j.1398-9995.2011.02770.x 22235793

[2] Asher MI Montefort S Björkstén B Lai CK Strachan DP Weiland SK Worldwide time trends in the prevalence of symptoms of asthma, allergic rhinoconjunctivitis, and eczema in childhood: ISAAC Phases One and Three Repeat Multicountry Cross-Sectional Surveys. Lancet 2006 368 733 43 10.1016/S0140-6736(06)69283-0 16935684

[3] Burney P Malmberg E Chinn S Jarvis D Luczynska C Lai E The distribution of total and specific serum igE in the European Community Respiratory Health Survey. J Allergy Clin Immunol 1997 99 314 22 10.1016/S0091-6749(97)70048-4 9058686

[4] Peternel R Toth I Hercog P Vojniković B Cop R Bradić-Hammoud M Influence of aeroallergens on the incidence of conjunctivitis in Zagreb and Zagreb County. Coll Antropol 2013 37 Suppl 1 13 7 23837216

[5] Toth I Peternel R Gajnik D Vojniković B Micro-regional hypersensitivity variations to inhalant allergens in the city of Zagreb and Zagreb County. Coll Antropol 2011 35 Suppl 2 31 7 22220400

[6] Pawankar R, Canonica GW, Holgate S, Lockey R, editors. World Allergy Organization (WAO) white book on allergy: Update 2013; World Allergy Organization, 2013.

[7] Zuberbier T Lötvall J Simoens S Subramanian SV Church MK Economic burden of inadequate management of allergic diseases in the european Union: A GA 2 LEN Rev Allergy 2014 69 1275 9 10.1111/all.12470 24965386

[8] Beutner C Werchan B Forkel S Gupta S Fuchs T Schön MP Sensitization rates to common inhaled allergens in Germany – increase and change patterns over the last 20 years. J Dtsch Dermatol Ges 2021 19 37 44 10.1111/ddg.14312 33103355

[9] Topalušić I Stipić Marković A Artuković M Dodig S Bucić L Lugović Mihić L Divergent trends in the prevalence of children’s asthma, rhinitis and atopic dermatitis and environmental influences in the urban setting of Zagreb, Croatia. Children (Basel) 2022 9 1788 36553232 10.3390/children9121788 PMC9777289

[10] Carlson G Coop C Pollen food allergy syndrome (PFAS): A review of current available literature. Ann Allergy Asthma Immunol 2019 123 359 65 10.1016/j.anai.2019.07.022 31376490

[11] Bousquet J Melén E Haahtela T Koppelman GH Togias A Valenta R Rhinitis associated with asthma is distinct from rhinitis alone: The ARIA-MeDALL Hypothesis. Allergy 2023 78 1169 203 10.1111/all.15679 36799120

[12] Simons FE Allergic rhinobronchitis: the asthma-allergic rhinitis link. J Allergy Clin Immunol 1999 104 534 40 10.1016/S0091-6749(99)70320-9 10482824

[13] Global Initiative for Asthma, 2023. Global strategy for asthma management and prevention (2023 Update). Accessed: 1. December 2023.

[14] Bousquet J Schünemann HJ Togias A Bachert C Erhola M Hellings PW Next-generation allergic rhinitis and its impact on asthma (ARIA) guidelines for allergic rhinitis based on Grading of Recommendations Assessment, Development and Evaluation (GRADE) and real-world evidence. J Allergy Clin Immunol 2020 145 70 80.e3 10.1016/j.jaci.2019.06.049 31627910

[15] Global Initiative for Chronic Obstructive Lung Disease. Global strategy for the diagnosis, management, and prevention of chronic obstructive pulmonary disease (2024 Report). Available from: https://goldcopd.org/2024-gold-report/*.* Accessed: December 1, 2023.

[16] Skypala IJ Hunter H Krishna MT Rey-Garcia H Till SJ Du Toit G BSACI Guideline for the Diagnosis and Management of Pollen Food Syndrome in the UK. Clin Exp Allergy 2022 52 1018 34 10.1111/cea.14208 35975576

[17] Melén E Bergström A Kull I Almqvist C Andersson N Asarnoj A Male sex is strongly associated with IgE-sensitization to airborne but not food allergens: results up to age 24 years from the BAMSE Birth Cohort. Clin Transl Allergy 2020 10 15 10.1186/s13601-020-00319-w 32489587 PMC7247167

[18] Haftenberger M Laußmann D Ellert U Kalcklösch M Langen U Schlaud M (Prevalence of sensitisation to aeraoallergens and food allergens: results of the German Health Interview and Examination Survey for Adults (DEGS1)). Bundesgesundheitsblatt Gesundheitsforschung Gesundheitsschutz 2013 56 687 97 10.1007/s00103-012-1658-1 23703487

[19] Ahmed H Ospina MB Sideri K Vliagoftis H Retrospective analysis of aeroallergen’s sensitization patterns in Edmonton, Canada. Allergy Asthma Clin Immunol 2019 15 6 10.1186/s13223-019-0320-y 30809266 PMC6375155

[20] Pinart M Keller T Reich A Fröhlich M Cabieses B Hohmann C Sex-related allergic rhinitis prevalence switch from childhood to adulthood: A systematic review and meta-analysis. Int Arch Allergy Immunol 2017 172 224 35 10.1159/000464324 28456795

[21] Beutner C Forkel S Gupta S Fuchs T Schön MP Geier J Sex- and age-dependent changes in polysensitization to common aeroallergens over 20 years. J Asthma Allergy 2020 13 725 30 10.2147/JAA.S280771 33390729 PMC7772690

[22] Warm K Backman H Lindberg A Lundbäck B Rönmark E Low incidence and high remission of allergic sensitization among adults. J Allergy Clin Immunol 2012 129 136 42 10.1016/j.jaci.2011.08.033 21975174

[23] Chen H Johnson CA Haselkorn T Lee JH Israel E Subspecialty differences in asthma characteristics and management. Mayo Clin Proc 2008 83 786 93 10.4065/83.7.786 18613995 PMC3102298

[24] Romanet-Manent S Charpin D Magnan A Lanteaume A Vervloet Dand the EGEA Cooperative Group allergic vs nonallergic asthma: what makes the differenceAllergy 2002 57 607 13 10.1034/j.1398-9995.2002.23504.x 12100301

[25] Blanc PD Katz PP Henke J Smith S Yelin EH Pulmonary and allergy subspecialty care in adults with asthma. treatment, use of services, and health outcomes. West J Med 1997 167 398 407 9426478 PMC1304718

[26] Shargorodsky J Garcia-Esquinas E Galán I Navas-Acien A Lin SY Allergic sensitization, rhinitis and tobacco smoke exposure in US adults. PLoS One 2015 10 e0131957 10.1371/journal.pone.0131957 26172447 PMC4501790

[27] Özdemir E Damadoğlu E Karakaya G Kalyoncu AF Prevalence and clinical features of pollen-food allergy syndrome in adults with seasonal allergic rhinitis. Eur Rev Med Pharmacol Sci 2023 27 103 9 36647856 10.26355/eurrev_202301_30858

[28] Vieru M, Popescu F-D, Tudose A, Dumitrescu N. Sensitisation pattern to birch pollen allergen components in oral allergy syndrome to rosaceae fruits in patients with spring pollinosis from an East European Sylvosteppe area with low density forests. Clin Transl Allergy. 2014;4:P47, 2045-7022-4-S2-P47.

[29] Treudler R Zeynalova S Kirsten T Engel C Loeffler M Simon JC Living in the city centre is associated with type 1 sensitization to outdoor allergens in Leipzig, Germany. Clin Respir J 2018 12 2686 8 10.1111/crj.12967 30266039

[30] Elholm G Linneberg A Husemoen LLN Omland Ø Grønager PM Sigsgaard T The Danish urban-rural gradient of allergic sensitization and disease in adults. Clin Exp Allergy 2016 46 103 11 10.1111/cea.12583 26096697

[31] Antić N.Kretanje stanovništva Grada Zagreba s posebnim osvrtom na doseljavanje u razdoblju 1991Migr Etn Teme 2001 17 287 309

[32] Galzina N Barić K Šćepanović M Goršić M Ostojić Z Distribution of invasive weed Ambrosia Artemisiifolia L. in Croatia. ACS Agric Conspec Sci 2010 75 75 81

[33] Peternel R Culig J Srnec L Mitić B Vukusić I Variation in Ragweed (Ambrosia Artemisiifolia L.) pollen concentration in Central Croatia, 2002-2003. Ann Agric Environ Med 2005 12 11 6 16028859

[34] Heinzerling L Frew AJ Bindslev-Jensen C Bonini S Bousquet J Bresciani M Standard skin prick testing and sensitization to inhalant allergens across Europe – a survey from the GA 2 LEN Network*. Allergy 2005 60 1287 300 10.1111/j.1398-9995.2005.00895.x 16134996

[35] Forkel S Beutner C Heetfeld A Fuchs T Schön MP Geier J Allergic rhinitis to weed pollen in Germany – dominance by plantain, rising prevalence, and polysensitization rates over 20 years. Int Arch Allergy Immunol 2020 181 128 35 10.1159/000504297 31805564

[36] Burbach GJ Heinzerling LM Edenharter G Bachert C Bindslev-Jensen C GA 2 LEN Skin Test Study II: clinical relevance of inhalant allergen sensitizations in Europe. Allergy 2009 64 1507 15 10.1111/j.1398-9995.2009.02089.x 19772516

[37] Lyons SA Clausen M Knulst AC Ballmer-Weber BK Fernandez-Rivas M Prevalence of food sensitization and food allergy in children across Europe. J Allergy Clin Immunol Pract 2020 8 2736 2746.e9 10.1016/j.jaip.2020.04.020 32330668

[38] Sekerková A Poláčková M Detection of Bet v1, Bet v2 and Bet v4 Specific IgE antibodies in the sera of children and adult patients allergic to birch pollen: evaluation of different igE reactivity profiles depending on age and local sensitization. Int Arch Allergy Immunol 2011 154 278 85 10.1159/000321819 20962532

[39] Loraud C de Ménonville CT Bourgoin-Heck M Cottel N Wanin S Just J Emergence of pollen food allergy syndrome in asthmatic children in Paris. Pediatr Allergy Immunol 2021 32 702 8 10.1111/pai.13435 33332662

